# Delineation in thoracic oncology: a prospective study of the effect of training on contour variability and dosimetric consequences

**DOI:** 10.1186/1748-717X-6-118

**Published:** 2011-09-19

**Authors:** Sylvain Dewas, Jean-Emmanuel Bibault, Pierre Blanchard, Claire Vautravers-Dewas, Yoann Pointreau, Fabrice Denis, Michel Brauner, Philippe Giraud

**Affiliations:** 1 Département universitaire de Radiothérapie, Centre Oscar Lambret, Université Lille II, Lille, France; 2 Société Française des jeunes Radiothérapeutes-Oncologues (SFjRO), Paris, France; 3 Département Universitaire de Radiothérapie, Institut Gustave Roussy, Villejuif, France; 4 Département universitaire de Radiothérapie, Centre Georges François Leclerc, Dijon, France; 5 Clinique d'Oncologie radiothérapie, Centre Henry S Kaplan, CHU Bretonneau, Tours, France; 6 Association pour la Formation Continue en Oncologie-Radiothérapie (AFCOR), Paris, France; 7 Service de radiologie, Université Paris 13, Hôpital Avicenne, Assistance Publique-Hôpitaux de Paris, Bobigny, France; 8 Université Paris Descartes, service d'Oncologie Radiothérapie, Hôpital Européen Georges Pompidou, Paris, France

**Keywords:** Lung cancer, volume delineation, education, conformal radiotherapy, inter-observer variability

## Abstract

**Introduction:**

As part of French residents' radiotherapy training, delineation workstations were available at a national teaching course. We report a prospective comparative study of a non small cell lung cancer (NSCLC) case delineated by 120 residents before and after a radioanatomy/radiotherapy lecture.

**Materials and methods:**

The case of a patient with right upper lobe non small cell lung cancer (NSCLC) was provided for delineation to 32 groups of residents before and after a radiation therapy lecture about thoracic delineation. GTV, CTV and PTV was asked to each group. In a second step, the GTV, CTV and PTV were compared with those of 9 groups of senior physicians. Finally the consequences for treatment planning between each group before and after the course were explored.

**Results:**

The expert's average GTV, CTV and PTV were 89.1 cm^3^, 242.3 cm^3 ^and 293.9 cm^3 ^respectively. For residents, those volumes were 103.4 cm^3^, 242.3 cm^3 ^and 457.9 cm^3 ^before teaching, compared to 99.5 cm^3^, 224.2 cm^3 ^and 412.5 cm^3 ^after teaching. The overlap (OV) and kappa (KI) indices before and after education were respectively 0.58 and 0.73. Compared to senior physicians, OV and KI indices were lower in the residents group (p = 0.039 and p = 0.043). An increased dose to the lung is noted for the residents' dosimetry compared to the experts' (V20: 23.2% versus 36.5%) due to the larger PTV delineated. No significant difference was observed for other organs at risk.

**Conclusion:**

There were no significant differences for the delineation of the GTV and CTV before and after the course, although the differences tended to decrease after the course. The good initial quality of the contours could explain the lack of difference. V20 for lung was higher in the residents group compared to the experts group (23.2% vs 36.5%). No other treatment planning consequences were observed for other critical organs.

## Introduction

The variability among radiation-oncologists in the delineation of GTV for lung cancer has already been described [1-4]. These variations are a major source of error in the planning and execution of radiotherapy. However, few studies are available regarding the effect of training of radiotherapy residents on this variability [5]. This question is pertinent at the moment because of recent developments in radiation therapy using optimized radiation dose distributions, which allow greater precision in delivering and repositioning in order to increase the total dose delivered to the target volume [6-8]. Consequently, the delineation of the different target volumes should have the same precision as these new technologies. Inaccurate contouring leads to incorrect dosimetry, and poor quality treatment results in lower tumor control and increased toxicity [3]. During the national training of French radiotherapy residents, a comparison of residents' delineation of the same clinical case of non small cell lung cancer (NSCLC) was proposed, before and after a teaching course on radioanatomy and radiotherapy techniques of lung cancer irradiation. We evaluated the influence of the teaching course on the variability of the residents' outlines and their conformity to a "reference" delineation (corresponding to the clinically used contours). We then analyzed the dosimetric effect of the variability. The same clinical case had also been delineated in a previous session by senior physicians whose delineations have also been compared with the residents' ones.

## Methods

### Objectives

The objective of this study was to search for differences in delineation between the residents and the experts before and after a radiation therapy course about thoracic volumes definition. Volumes (GTV, CTV and PTV), indices (VR, CDV, ADV, KI and OV) and treatment planning were compared.

### Population

One hundred and twenty eight French residents attended a national course on radioanatomy organized in Paris at the European Hospital Georges Pompidou in 2009. These courses are held every four years to teach new imaging techniques and different delineation recommendations for the main tumor locations. For the first time, 8 delineation workstations were made available for practice sessions on real clinical cases. The 128 residents were divided into 32 mixed groups of 4 (31, 25, 28, 19 and 17 residents respectively in 5th, 4th, 3rd, 2nd and 1st year of training). In order to evaluate the effect of training with respect to the delineation recommendations, a clinical case of NSCLC was chosen. This case represents a common location with many, and typical, technical difficulties for residents.

The same clinical case was previously contoured under similar conditions by 9 groups of senior radiation oncologists during a training course organized by the Association for Continuing Education in Oncology-Radiotherapy (AFCOR, Association de Formation Continue en Oncologie Radiothérapie). These results were compared with those of the residents.

### Clinical case presentation

**Figure 1:**
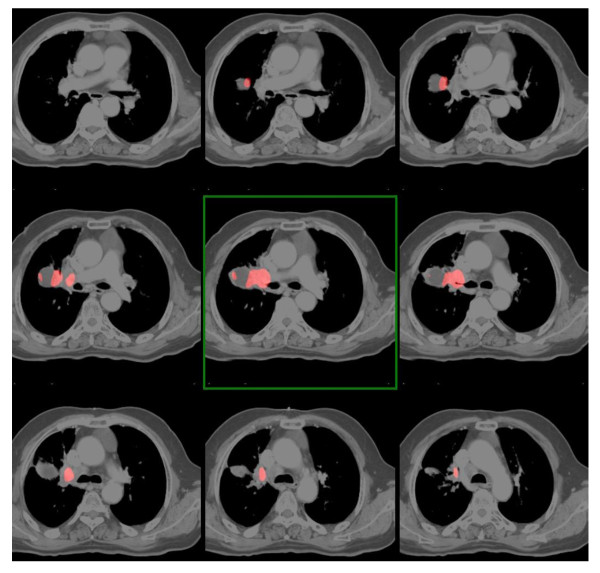
**Clinical case available to attendees**. CT Fusion with PET. Three groups included a volume without FDG uptake inside the GTV even though it had been explained before delineation that it was considered as a thymic residue.

The clinical case involved a male, age 67, with tobacco consumption estimated at 40 pack-years, no medical or surgical history, and no asbestos exposure. The evaluation of a chronic progressive cough persisting > 3 months demonstrated an opacity in the right upper lobe. Bronchoscopy found a stenosing lesion in the right upper lobe bronchus and biopsy samples revealed a squamous cell carcinoma. The CT scan found a voluminous chest lesion measuring 64 mm in diameter at the origin of the right upper lobe. Multiple nodes were found in Barety's space, the pre-aortic space, and the left internal mammary nodes (Figure 1). Overall, it was classified T2N3M0. After three cycles of chemotherapy combining Cisplatin and Vinorelbine, concomitant radio-chemotherapy with the same chemotherapy and a deep inspiration breath hold technique (SDX, Dyn'R, Muret, France) was proposed to the patient because of the tumor stability. A pre-chemotherapy PET-CT fusion was available (Figure 1). Contrast enhancement was used for the treatment planning CT Scan, without breathhold or 4D.

### Education and delineation

This case was presented and delineated by students before and after a theoretical course provided by two lung cancer experts. The first one-hour lecture, conducted by a senior radiologist, covered radio-thoracic anatomy (MB). The second one-hour lecture, taught by a senior radiotherapist, also dealt with irradiation techniques, with emphasis on the recommended volumes, processing techniques, and dose and margins to respect; all recommendations were based on the literature (PG). No discussion about the clinical case presented was made during the lectures by any of the two teachers. The delineation was made independently by each group of students with ARTIVIEW™ software (AQUILAB^®^, Lille, France). The initial diagnostic CT scan, the initial PET-CT scan and the PET fusion with the planning CT scan were available to the residents. The software employs standard delineation tools (pen, brush, multislice interpolation...). The residents could modify the image contrast and sagittal or coronal reconstructions as needed.

For each delineation session, the residents were asked to draw the GTV on the CT scan. Each group then had to prescribe margins to the GTV (by 3D isotropic geometric expansion) in order to obtain the clinical target volume (CTV), and then the planning target volume (PTV). The prescribed dose to the PTV was also collected. Among the 128 residents registered, 120 students participated in the two contouring sessions. Thirty evaluable groups were compared before and after the course. Each group consisted in four residents from different training years in order to have homogeneous groups. Delineation practice lasted 15 minutes for each group.

### Methods of comparison

#### I - Volume comparison

**Figure 2:**
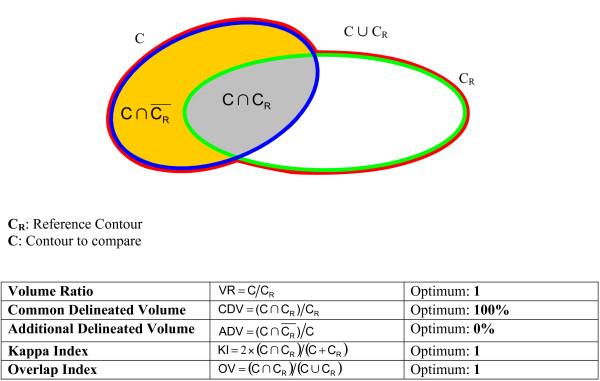
**Volumes: calculated indices**.

The evaluation of the variability of contours was benchmarked using a "reference" contour (delineated by the expert who provided the clinical case PG). ARTIVIEW™ software was used to evaluate and compare the contours, both qualitatively and quantitatively. For the first step, each target volume submitted by each group of students (GTV, CTV and PTV) was compared with the reference volume proposed by the expert. In the second step, different indices reflecting the correlation of the volume with the "expert reference contour" for all volumes were calculated [9-12]. The CTV was obtained by a geometric expansion of the GTV and the PTV was a geometric expansion of the CTV. Groups had no instructions regarding the size of the margins to apply. The observers were not allowed to adapt the CTV to the natural anatomical borders of tissue,

The first family of indices is based on volume ratios: the volume ratio (VR) between the volume of the student and the volume of the expert, the common delineated volume (CDV) and the additional delineated volume (ADV). Then, the kappa index (KI) and the overlap volume (OV) were calculated (Figure 2). The last step consisted of an analysis of variance between pre and post-course delineated volumes.

#### II - Treatment planning comparison: The dose was calculated using the Oncentra^® ^Collapsed Cone algorithm

**Figure 3:**
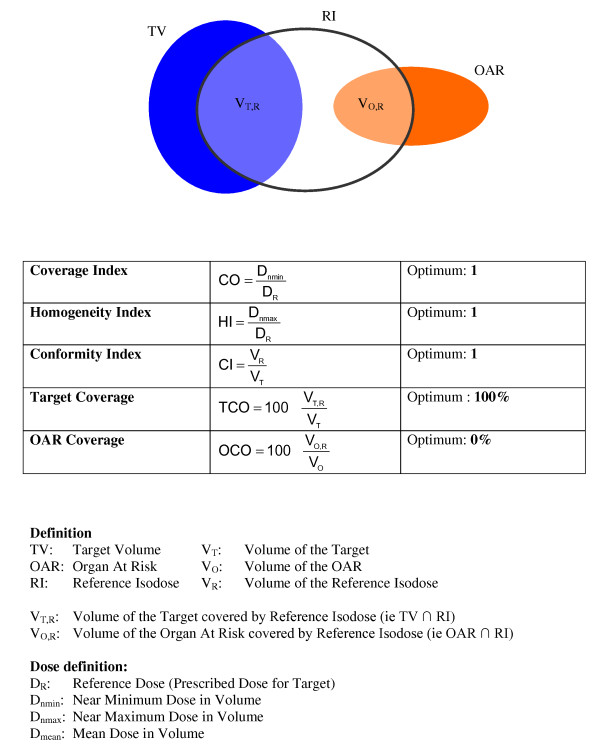
**Dose: calculated indices**.

The dosimetric effect of this variation in delineation was also analyzed. The group with the lowest conformal indices (KI and OV) at the end of the course, i.e. the most divergent, was studied and a treatment plan was calculated for this group. This plan was then compared with the treatment plan developed with the reference contours. The calculated dosimetric indices were the RTOG coverage index (CO), the RTOG homogeneity index (HI), the RTOG conformity index (CI), the target coverage index (TCO) and the organs at risk (OAR) coverage index (OCO) [9-12]. The CO Index is the ratio of the minimum dose delivered in 98% of the volume of interest and the reference prescribed dose (Figure 3).

#### III - Comparison with senior physicians

In the last step, the GTV outlines (before and after the course) made by residents were compared to the GTV delineated by senior physicians during a previous session on lung cancer organized by AFCOR. This delineation had been done under exactly the same conditions, with the same tools and the same clinical information as the residents; the contours were done after a training session on lung radiation similar to that taught to residents (PG). The same indices were calculated in order to compare the GTV of residents and senior physicians.

### Statistical Analysis

Delineation evaluation was conducted prospectively and comparatively. Statistical analysis was performed with SPSS 13.0 (SPSS. Inc., Chicago, Ill., USA). Comparison between the volume obtained (residents and senior physicians) and the expert's volume was done with the Student t-test. Comparative analysis of indices before and after the course was done with the Student t-test for paired samples. The variance of the different volumes was compared by ANOVA. A *P-*value < .05 was considered statistically significant.

## Results

### I - Volume comparison

**Figure 4:**
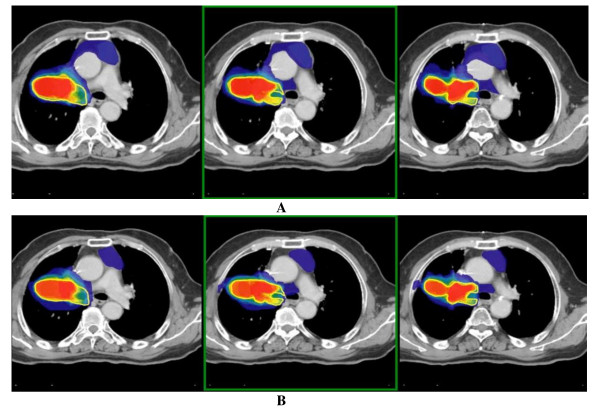
**GTV inter-comparison before (A) and after the course (B) (red: 30 groups, blue: 3 groups, yellow contour: expert's contour)**.

The GTV delineated by the expert was measured at 89.1 cm^3 ^and extended 6.5 cm on the vertical plane. The average GTV volume delineated by the students before the course was 103.4 cm^3 ^(range of 59.9 to 215.2 cm^3^), significantly different from that of the expert (p = 0.02). After the course, it was 99.5 cm^3^, not significantly different from that of the expert (39.7 to 202.3, p = 0.09) (Figure 4). The average height of the GTV contoured by residents was 5.8 cm (3.5 to 6.5 cm) before the course and 5.9 cm (4.5 to 8 cm in height) after the course (NS). The mass in the left internal mammary nodes was not considered to be pathological by the expert because there was no PET fixation. This opinion had been explained during the course by the expert; it was considered possible residual thymic tissue. Three groups incorrectly delineated the residual thymic tissue before the training and only one group after training.

The CTV was obtained by a geometric expansion of the GTV. The median margin required by the students was 6 mm prior to the course (5 to 10 mm) and was 6 mm (5 to 6 mm) after the course. The expansion margin required by the expert was 6 mm.^13 ^The volume of the CTV obtained by the expert was 214.3 cm^3^. The average volume of the CTV for the residents was 242.3 cm^3 ^(162.9 to 495.7 cm^3^) before the course. After the course, the average volume of CTV was 224.2 cm^3 ^(110.3 to 385.2 cm^3^). The two volumes were not significantly different from the expert's (respectively p = 0.065 and p = 0.38) although the differences tended to decrease after the course.

The median margin required by students for the geometric expansion of the CTV to PTV was 7 mm prior to the course (3 to 15 mm) compared to 5 mm (3 to 15 mm) after the course. The expansion margin required by the expert was 5 mm, using a respiratory gating technique (deep inspiration breath hold). The PTV volume obtained by the expert was 293.9 cm^3^. The average PTV volume of the students before the course was 457.9 cm^3 ^(248.5 to 1011.3 cm^3^), different from the expert's (p < 0.001), compared to 412.5 cm^3 ^after the course (196.4 to 645.7 cm^3^), different from the expert's (p < 0.001).

In terms of the calculated indices, no significant difference was found before and after the course for GTV (table 1) or for CTV and PTV. The analysis of variance (ANOVA) before and after the course was not significantly different. This result was previously published in another study [13].

**Table 1:** Comparison indices for the GTV

			Comparison with the expert	Comparison before and after the course			
				
				Range		Mean Difference	P*
						
				Min	Max		
**GTV (cm^3^)**	Before	103.39	p = 0.02	59.9	215.2	3.90	0.53
			
	After	99.48	p = 0.09	39.71	202.29		

**Volume Ratio**	Before	1.16	p = 0.02	0.67	2.41	0.04	0.53
			
	After	1.11	p = 0.09	0.44	2.27		

**Common Delineated Volume**	Before	78.41	p < 0.001	58.85	91.78	2.06	0.27
			
	After	76.35	p < 0.001	40.24	92.06		

**Additional Delineated Volume**	Before	28.77	p < 0.001	7.05	61.99	0.94	0.70
			
	After	27.82	p < 0.001	9.14	59.56		

**Overlap**	Before	0.58	p < 0.001	0.36	0.71	0.006	0.70
			
	After	0.57	p < 0.001	0.38	0.71		

**Kappa**	Before	0.73	p < 0.001	0.54	0.83	0.006	0.66
			
	After	0.72	p < 0.001	0.56	0.83		

* (*t *test and paired *t *test).

### II - Effect of contour variability on the treatment plan

The expert recommended a dose of 66 Gy to the PTV. The mean dose prescribed by the residents before the course was 66.16 Gy (60 to 70 Gy) compared to 66.5 Gy (64 to 70 Gy) after the course. No significant difference was found. Two dosimetries were then constructed: the first using the contour of the expert, the second the contour from the group with the lowest indices of OV (0.39) and KI (0.56). The indices for the tumor obtained by the expert were 1.05 for the coverage index (optimum 1), 1.05 for homogeneity index (optimum 1), 0.58 for conformity index (optimum 1) and 54.1% for the target coverage index (optimum 100%). For the group of residents, they were respectively 1.04, 1.04, 0.51 and 45.6%. The treatment plan of the resident group with the lowest OV and KI indices was applied on the contours of the expert. Then, the different indices for the tumor became: 1.04, 1.04, 1 and 53%.

**Figure 5:**
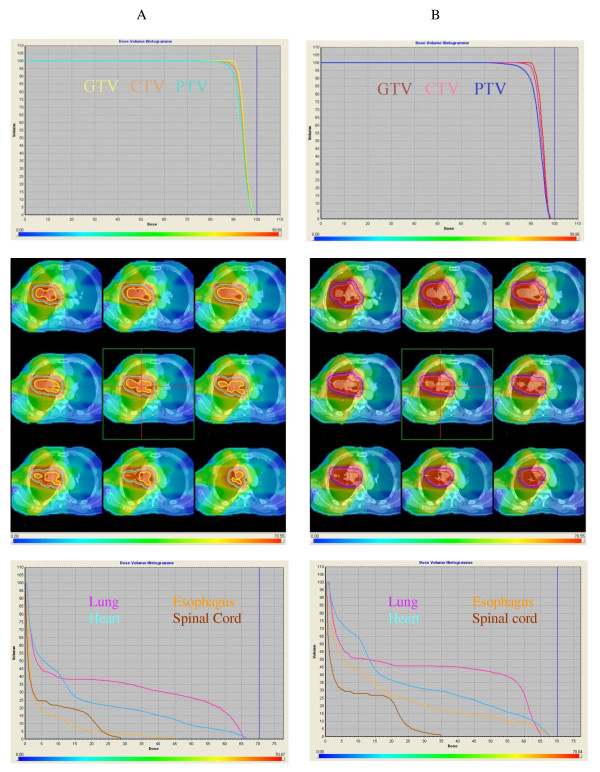
**Comparison of DVH between the expert (A) and the lowest overlap (0.39) and kappa (0.56) of the resident group (B)**.

The study of the dosimetric effect on organs at risk was also performed for both treatment plans. The dose-volume histograms are shown in Figure 5. The calculation for OCO (OAR coverage index) was made using a dose of 20 Gy to the lungs, 40 Gy to the heart, 55 Gy to the esophagus, and 45 Gy to the spinal cord. The OCO factor for the expert was 23.2% (max dose 66.6 Gy) to the lungs (i.e. V20 = 23.2%), 1.40% (max dose 44.5 Gy) to the heart, 23.3% (up to 65.7 Gy) to the esophagus and 0% (max dose 28.9 Gy) to the spinal cord. For the group of residents, it was respectively 36.5% (max dose 67.5 Gy), 15.4% (max dose 67.3 Gy), 38.9% (max dose 65.2 Gy) and 0% (max dose 34.9 Gy). An increased dose to the lung is noted for the residents' dosimetry (V20: 23.2% versus 36.5%) due to the larger PTV delineated. No significant difference was observed for other organs at risk.

### III - Comparison with senior physicians

Nine GTVs delineated by senior radiation oncologists were also available from a previous course. The average GTV volume was 96.9 cm^3 ^(68.5 to 124.6 cm^3^), not significantly different from the expert (p = 0.2). The different indices obtained by the seniors compared to the expert were for the volume ratio 1.08 (0.77 to 1.4, CI 95%: 0.93-1.24), 81.3% (69.7 to 88.7%, CI 95%: 76.2-86.3) for the common delineated ratio, 23.9% (9.4 to 36.7%, CI 95%: 17.4-30.4) for the additional delineated volume, 0.64 (0.58 to 0.71, CI 95%: 0.61-0.67) for the overlap volume and 0.78 (0.74 to 0.83, CI 95%: 0.76-0.8) for the kappa index. Comparison of volumes and GTV indices with those of residents before the course was: 96.9 cm^3 ^compared to 103.4 cm^3 ^(NS) for the volume of the GTV, 1.08 compared to 1.16 (NS) for the VR, 81.3% compared to 78.4% (NS) for the CDV, 23.9% compared to 28.8% (NS) for the ADV, 0.64 compared to 0.58 (p = 0.045) for the OV, and 0.78 compared to 0.73 (p = 0.049) for the KI. After the course, the GTV was 99.5 cm^3 ^for the residents compared to 96.9 cm^3 ^for the senior physicians (NS). The difference between residents and physicians for OV and KI was 0.06 (p = 0.047) and 0.05 (p = 0.05). No significant difference was noted for the VR, CDV and ADV. The global OV and KI indices (mean before and after the course) compared to the senior physician's delineation were always lower in the residents group (respectively, p = 0.039 and p = 0.043). The study of the distribution showed a lower variance among physicians than among residents for OV and KI (p = 0.039 and p = 0.043). Residents had, more frequently than senior physicians, an OV under 0.6 (p = 0.03), and senior physicians had significantly more often a KI greater than 0.75 (p = 0.03).

## Discussion

This study showed a non-significant trend in improved delineations after a teaching course among residents. However, the very high quality of the initial delineations could explain the lack of significant progress after teaching. Another explanation for this could be the short period of time available for each delineation (15 minutes) that could limit the variations of volumes. Moreover, the fact that several residents discussed together in order to reach an agreement, inside their groups, about the volumes that needed to be treated could explain the homogeneity and the good quality of the contours. One could think that a single resident/group would increase errors thus decreasing the overall quality of the contours. Another possible explanation would be that residents were not allowed to adapt the CTV to the natural anatomical borders of tissue, decreasing even more the variability between observers.

The GTV delineation tended to decrease at the end of the course, from an initial 103.4 to 99.49 cm^3^. The difference compared to the expert's delineation was significant prior to the course (p = 0.02), but was no longer found after the course, reflecting an improvement in the residents' delineation. This decrease in volume was also found for the PTV (457, compared to 412 cm^3 ^after the course), linked both to the decrease of the delineated GTV volume and the margin expansion prescribed by the residents. The reduction of these margins could be related to the course, which clarified the delineation using matched CT-PET, and addressed the principles and advantages of the treatment technique (respiratory gating) and better knowledge of thoracic anatomy [3,14]. Guidelines in lung cancer delineation have long been known [15-17]. Lung cancer, like head and neck, is often used as an example in teaching. These guidelines are often used by residents during their practice training, which could explain the very high quality of the initial delineation.

We have previously demonstrated, using the delineation of 9 radiologists and 8 radiation oncologists, that resident physicians, regardless of their specialty, also tend to delineate smaller and more homogeneous volumes than senior physicians, especially for 'difficult' cases [3]. In order to confirm these results, a comparison of delineated volumes between residents and senior physicians was conducted. The physicians' contours seemed better than those of residents, both for volumes and inter-comparison indices. The variance was also lower, indicating better reproducibility of the delineations, probably related to experience. The delineations made by residents after the theoretical course were closer to those of senior physicians, reflecting the necessity of training.

Delineation variations are a source of error in the planning and execution of radiotherapy.

Regarding the treatment planning consequences of the variability of the delineation, we found a difference for V20 for the lung between the experts and the residents (23.2% vs 36.5%). This difference could be explained by the margins applied to obtain the PTV (7 mm before vs 5 mm after the course). The subsequent PTV volume was higher before the course (457.9 cm^3 ^vs 412.5 cm^3^). Thus V20 was also higher with the contours created before the course. This result shows the importance of the margins applied to the CTV to create the PTV to better respect the dose constraints to the critical organs, the final aim of this being lower toxicity. However there were no differences found for the other critical organs (esophagus, heart and spinal cord).

Delineation variability has already been analyzed, in prostate cancer [18,19], breast cancer [20], lung cancer [15], and cervical cancer [21]. Optimizing delineation may be done in several ways. In clinical trials, clinical reference cases and a "virtual patient" (dummy run) are available for radiation-oncologists to standardize their delineation procedures. Quality assurance studies have been also carried out [22-24]. Another way to standardize delineation is the creation of atlas support, available online or on paper. More and more locations are referenced, including head-and-neck cancer [25], gynecological [26], prostate [19,27], and lung cancer [15,16]. Based on this principle, a delineation self-training website has also been introduced in France since October 2008 http://www.siriade.org. This website is supported by AFCOR and the French Radiation Society (SFRO, Société Française de Radiothérapie Oncologique). Therefore, the organization of seminars or training courses for residents perfectly fits this logic, combining delineation teaching techniques and quality control. These courses are held every four years and concern the main tumor locations. A study conducted by residents of Memorial Sloan-Kettering Cancer Center in New York on a head-and-neck cancer case was published in 2008 [5]. Eleven delineations were collected before and after a theoretical course. Delineation was improved after education. Our study also demonstrates that incorporating a practical delineation workshop during training improves residents' delineation. These two studies highlight the importance of practical and theoretical training in the curriculum of radiation oncology residents.

## Conclusion

There were no significant differences before and after the course in the delineation of the GTV or CTV, although the differences tended to decrease after the course. However a difference was observed for the PTV. V20 for lung was higher in the residents group compared to the experts group (23.2% vs 36.5%). No other treatment planning consequences were observed for other critical organs.

## Ethical Approval and Consent

Not applicable

## Competing interests

The authors declare that they have no competing interests.

## Authors' contributions

SD, CVD, PB, YP and PG conceived the study. SD collected data. SD and JEB drafted the manuscript. FD and MB participated in coordination and helped to draft the manuscript. SD performed the statistical analyses. PG provided mentorship and edited the manuscript. All authors have read and approved the final manuscript.
